# Single chest tube drainage is superior to double chest tube drainage after lobectomy: a meta-analysis

**DOI:** 10.1186/s13019-016-0484-1

**Published:** 2016-05-27

**Authors:** Dong Zhou, Xu-Feng Deng, Quan-Xing Liu, Qian Chen, Jia-Xin Min, Ji-Gang Dai

**Affiliations:** Department of Thoracic Surgery, Xinqiao Hospital, Third Military Medical University, Chongqing, 400037 China; Institute of Pathology and Southwest Cancer Center, Southwest Hospital, Third Military Medical University, Chongqing, 400037 China

**Keywords:** Chest tube, Lobectomy, Drainage, Pain, Complications

## Abstract

**Background:**

In this meta-analysis, we conducted a pooled analysis of clinical studies comparing the efficacy of single chest tube versus double chest tube after a lobectomy.

**Methods:**

According to the recommendations of the Cochrane Collaboration, we established a rigorous study protocol. We performed a systematic electronic search of the PubMed, Embase, Cochrane Library and Web of Science databases to identify articles to include in our meta-analysis. A literature search was performed using relevant keywords. A meta-analysis was performed using RevMan© software.

**Results:**

Five studies, published between 2003 and 2014, including 630 patients (314 patients with a single chest tube and 316 patients with a double chest tube), met the selection criteria. From the available data, the patients using a single tube demonstrated significantly decreased postoperative pain [weighted mean difference [WMD] −0.60; 95 % confidence intervals [CIs] −0.68–− 0.52; *P* < 0.00001], duration of drainage [WMD −0.70; 95 % CIs −0.90–− 0.49; *P* < 0.00001] and hospital stay [WMD −0.51; 95 % CIs −0.91–− 0.12; *P* = 0.01] compared to patients using a double tube after a pulmonary lobectomy. However, there were no significant differences in postoperative complications [OR 0.91; 95 % CIs 0.57–1.44; *P* = 0.67] and re-drainage rates [OR 0.81; 95 % CIs 0.42–1.58; *P* = 0.54].

**Conclusion:**

Our results showed that a single-drain method is effective, reducing postoperative pain, hospitalization times and duration of drainage in patients who undergo a lobectomy. Moreover, the single-drain method does not increase the occurrence of postoperative complications and re-drainage rates.

## Background

Intercostal chest drains are a routine component of the management of the pleural space after intrathoracic surgery. These drains are mainly used to remove liquid or air from the pleural space. The conventional method of pleural drainage after a thoracotomy or a lobectomy is the use of double chest drains placed in the apical and basal positions before closure [1, 2]. Although these drains are effective and widely accepted, they are painful for the patients, particularly during their removal.

In 2003, the ‘best evidence topic’ in The Annals of Thoracic Surgery addressed whether one- or two-tube chest drains in patients undergoing a lobectomy reduced postoperative pain [3]. The first study suggested that single chest drains may be superior to the conventional double chest drains in terms of patient tolerability and cost-effectiveness, as well as applicability to thoracic surgery with no disadvantages compared with the rigid chest drain. Theoretically, single tube chest drainage is easier to insert and causes less pain and discomfort for the patient during both the insertion and while the tube is in the chest compared with double tube drainage, but single tube drainage has the possibility of inadequate chest drainage. Although some recent studies have compared the effectiveness of the two methods, there are no available data to support which of these treatments is more effective, and there are no evidence-based consensus recommendations for the optimal chest tube method to be used in pulmonary lobectomy [4–7].

The objective of this meta-analysis was to conduct a pooled analysis of clinical studies to compare objective (duration of drainage, hospital stay, re-drainage rate and complications) and subjective (postoperative pain) outcomes with a single chest tube compared with a double chest tube in patients who underwent a pulmonary lobectomy.

## Methods

A rigorous study protocol was established according to the recommendations of the Cochrane Collaboration. Prior to the analysis, to ensure the highest quality for this meta-analysis, all of the objectives, inclusion and exclusion criteria, primary and secondary outcomes, and methods of synthesis were prespecified.

Two investigators independently searched MEDLINE, EMBASE, Web of Science and the Cochrane Library database CENTRAL. These databases were searched between May 7, 2015 and May 16, 2015. The search terms ‘lobectomy’, ‘chest tube’, ‘drainage’, ‘single’ and ‘double’ as well as the MeSH headings ‘lobectomy’ (MeSH), ‘chest tube’ (MeSH), ‘drainage’ (MeSH) ‘single’(MeSH) and ‘double’ (MeSH) were used in combination with the Boolean operators AND or OR. Studies were included if they met each of the following criteria: comparative studies and separation into groups based on the use of a single chest tube or a double chest tube after a lobectomy. Importantly, no attempt was made to search for unpublished literature, and studies published solely in foreign languages were excluded.

The primary outcome measures for the meta-analysis were postoperative pain, length of hospital stay and duration of drainage. The secondary outcome measures for the meta-analysis were postoperative complications (pneumothorax, pleural empyema, wound infection, atelectasis and persistent air leak) and re-drainage rate. Data from eligible trials were entered into a computerized spreadsheet for analysis. The quality of each trial was assessed using the Jadad scoring system.

### Statistical analysis

Synchronized extraction results were pooled statistically as effect estimates in the meta-analyses. We estimated the odds ratios (OR) for dichotomous outcomes and the weighted mean difference (WMD) for continuous outcomes. The level of heterogeneity (level of variance) across studies was evaluated using I^2^ statistics. The fixed effect model was initially used to calculate the pooled HR, and the random-effects model would be used if the clinical characteristics and methodology were not identified to be of great difference. Forest plots were generated for each of the six outcomes using the Review Manager (RevMan©) Version 5.3.

## Results

### Characteristics of the included trials

**Fig. 1:**
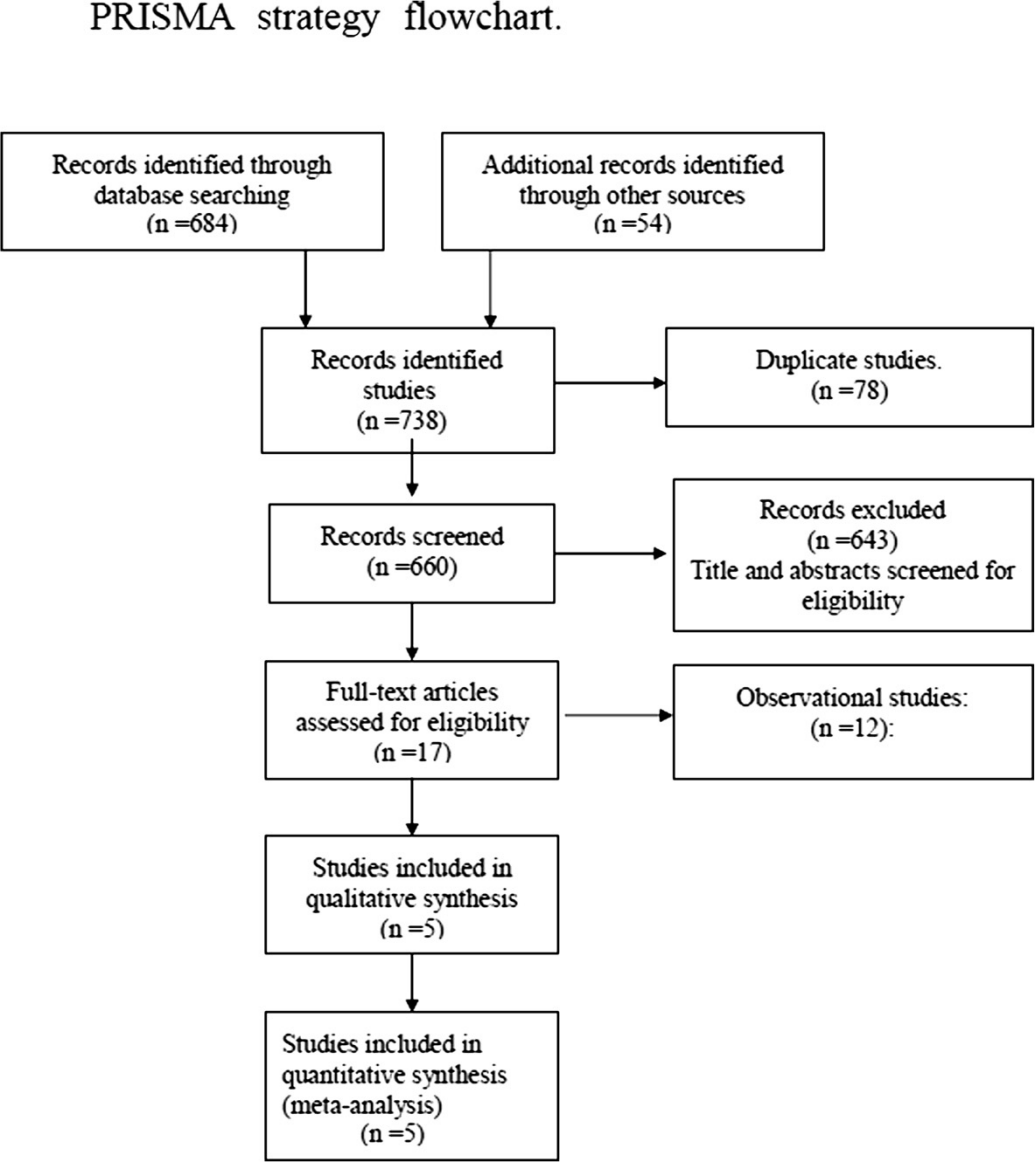
Flow chart of the literature search according to the PRISMA statement

The initial literature search yielded 738 citations, of which 5 studies were included (4 RCTs and 1 nRCTs) [3–7]. All eligible studies were published between 2003 and 2014. All cases included cancer patients. Table 1 shows the details for each trial, including baseline characteristics, publication year of the study, type of resection, and tumor stage for each trial. A PRISMA flowchart (Fig. 1) describes the details of the literature search for this systematic review.

**Table 1:** Demographic data

References	Publication year	Patients		Age (years)	Sex (male/female)	Type of resection	Tumor stage
Alex	2003	Group A	60	65 ± 8.4	48/12	Lobectomy	I–II
		Group B	60	66 ± 8.6	48/12		
Gomez-Caro	2006	Group A	60	65.5 ± 9.4	9/51	Lobectomy/Bilobectomy	I–IV
		Group B	59	61.5 ± 9.5	7/52		
Pawelczyk	2007	Group A	90	60.9 ± 9.03	64/26	Lobectomy/Bilobectomy	I–IV
		Group B	93	60.7 ± 8.906	54/39		
Okur	2009	Group A	50	54.74 ± 14.34	37/13	Lobectomy	NR
		Group B	50	56.34 ± 11.52	43/7		
Tanaka	2014	Group A	54	66.8 ± 7.5	38/16	Lobectomy/Bilobectomy	I–IV
		Group B	54	67.7 ± 8.0	32/22		

*Abbreviations*: *Group A* single chest tube, *Group B* double chest tube, *NR* not reported

### Postoperative pain

**Fig. 2:**
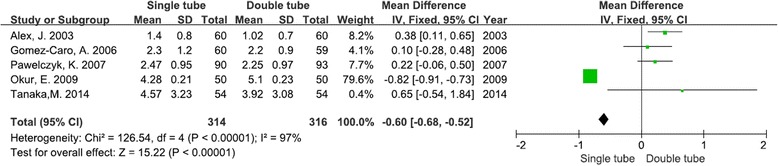
Forest plot for postoperative pain

Postoperative pain was measured in all of the five studies, totaling 314 patients with a single chest tube and 316 patients with a double chest tube. Our meta-analysis found that the use of only one drain was less painful for patients after pulmonary lobectomy [weighted mean difference (WMD) −0.60; 95 % confidence intervals (CIs) −0.68–− 0.52; *P* < 0.00001]. Heterogeneity was found to be significant. (*I*^2^ = 97 %, *χ*^2^ = 126.54, *df* = 4, *P* < 0.00001) (Fig. 2).

### Duration of drainage

**Fig. 3:**
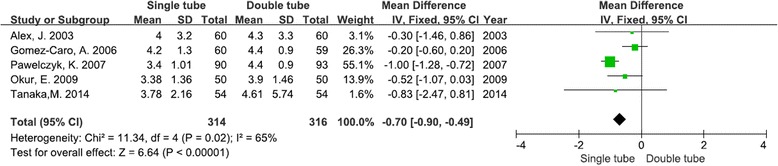
Forest plot for duration of drainage

All five of the studies reported the chest tube duration. Our meta-analysis found that patients with a single chest tube had their chest tubes removed sooner, and this finding was statistically significant [WMD −0.70; 95 % CIs −0.90–− 0.49; *P* < 0.00001]. Heterogeneity was found to be significant. (*I*^2^ = 65 %, *χ*^2^ = 11.34, *df* = 4, *P* = 0. 02) (Fig. 3).

### Length of hospital stay

**Fig. 4:**
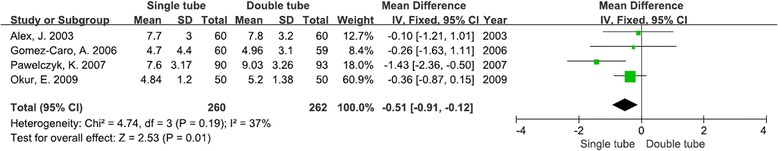
Forest plot for length of hospital stay

Four studies reported the length of the hospital stay. Patients with a single chest tube had a shorter length of stay, and this difference was statistically significant [WMD −0.51; 95 % CIs −0.91–− 0.12; *P* = 0.01]. There was no evidence of statistical heterogeneity (*I*^2^ = 37 %, *χ*^2^ = 4.74, *df* = 3, *P* = 0.19) (Fig. 4).

### Postoperative complications

**Fig. 5:**
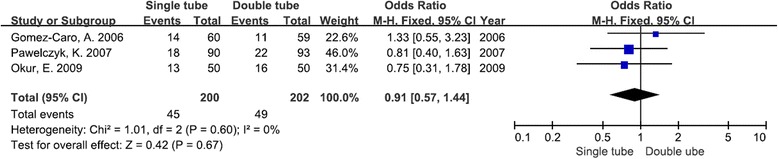
Forest plot for postoperative complications

The postoperative complications were available from three studies. The use of a single chest tube method does not increase the risk of postoperative complications in comparison with using the double chest tube method [odds ratio (OR): 0.91; 95 % CIs 0.57–1.44; *P* = 0.67]. There was no evidence of statistical heterogeneity (*I*^2^ = 0 %, *χ*^2^ = 1.01, *df* = 2, *P* = 0.60) (Fig. 5).

### Re-drainage rate

**Fig. 6:**
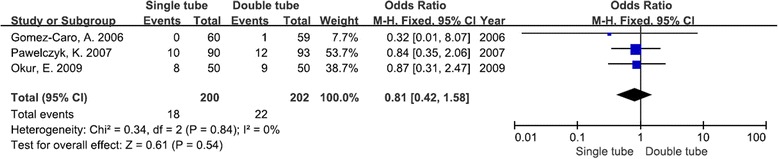
Forest plot for re-drainage rate

From the same three studies, it was found that there was no significant difference in the re-drainage rate between patients treated with a single chest tube or a double chest tube [OR 0.81; 95 % CIs 0.42–1.58; *P* = 0.54]. Statistical heterogeneity was not detected (*I*^2^ = 0 %, *χ*^2^ = 0.34, *df* = 2, *P* = 0.84) (Fig. 6).

## Discussion

One of the most common complications after a lobectomy is the inadequate re-expansion of residual lung [8]. To avoid this problem, the classical and widely accepted practice has been to place two drains for complete drainage of the pleural cavity after a lobectomy. One tube was placed in the midaxillary line on the most dependent side of the hemithorax, and the second tube was placed through the anterior axillary line towards the apex [9].

In recent years, many thoracic surgeons have adopted thoracic drainage using a single chest tube sited in the mid-position cavity after a pulmonary lobectomy. The first use of a single drain after a lobectomy has been reported by Alex J et al. [3] in a nonrandomized study. They concluded that a single chest drain in the mid-position decreased postoperative pain compared to the conventional use of two drains after a lobectomy, but there was no significant difference in the length of stay, duration of drainage and postoperative complications with the use of either a single or two drains. Thus far, there have only been four other reports of randomized controlled trials comparing the efficacy of a single chest tube versus a double chest tube after a lobectomy [4–7]. The results of the randomized trials revealed that proper expansion of the residual lung could be achieved even with one chest tube. These reports also revealed that there were no significant differences in the length of stay, duration of drainage and postoperative complications, whereas the overall costs were clearly reduced.

As reported in this review, the vast majority of studies employed small sample sizes and lacked the statistical power needed to make a clear statement regarding the utility of the single-drain method. A meta-analysis, such as that performed in this study, is a potentially useful tool in this context because pooling data can result in a very powerful study, as opposed to the results obtained from smaller individual studies. The purpose of this meta-analysis was to obtain a sufficiently large sample from different studies to reveal a potential significant difference between a single chest tube and a double chest tube after a lobectomy in terms of postoperative pain, duration of drainage, hospital stay, re-drainage rates and postoperative complications.

Interestingly, despite the fact that there were no significant differences in all of the observed targets in the above studies, pooling data from a large number of patients in this meta-analysis revealed that the single-drain method decreased postoperative pain, hospital stay and duration of drainage in patients who underwent a lobectomy. However, the results of the re-drainage rate and postoperative complications showed no significant differences between a single chest tube and a double chest tube, which were consistent with the results of the majority of these four randomized trials.

The classical practice is to use two tubes after pulmonary resections. One tube, which is placed anteriorly and directed to the apex, drains the air, and the other tube, which is placed more posteriorly and inferiorly, drains the fluid^1^. Our data demonstrated that the single-drain method not only achieved the same purposes of draining both the air and fluid, but it is also more effective, particularly in postoperative pain, hospital stay and duration of drainage, which suggests that this treatment should be routine. Some surgeons propose that postoperative pain control plays a major role in the postoperative period. Optimizing pain control helps in early lung re-expansion through deep breathing exercises, better cough and expectoration of secretions, reducing the hospital stay and duration of drainage [9–13].

However, several limitations of the present study exist. First, this study has a limitation due to its sample size. We could not identify the effect modifiers, which may be attributed to the low statistical power. Second, only English language literature articles were considered for inclusion. If the search had been extended to include literature published in other languages, then it is possible that additional relevant trials may have been identified. A final limitation is the statistically significant heterogeneity between the studies that evaluated postoperative pain and duration of drainage in the meta-analysis. The causes of heterogeneity among the studies could be related to the inherent heterogeneity of subjective sensation. In addition, unexplained heterogeneity remained in the meta-analysis of duration of drainage. There may have been between-study heterogeneity because the I^2^ remained high in the sensitivity analysis, which was potentially due to 2 outliers.

## Conclusion

In conclusion, our results showed that the single-drain method is effective, reduces postoperative pain, hospitalization times and duration of drainage in patients who undergo a lobectomy. Moreover, it does not increase the occurrence of postoperative complications and re-drainage rates. According to the results of our study, a single drain should be considered in patients after lobectomy or bilobectomy in common clinical practice.
